# P-883. Descriptive single center study on hospital acquired gram negative bloodstream infection: epidemiology, susceptibilities and outcomes in a six-year period

**DOI:** 10.1093/ofid/ofae631.1074

**Published:** 2025-01-29

**Authors:** Sivanthini Palanivetpillai, Tanis Dingle, Stephanie W Smith

**Affiliations:** University of Alberta, Edmonton, Alberta, Canada; Alberta Precision Laboratories, Edmonton, Alberta, Canada; University of Alberta, Edmonton, Alberta, Canada

## Abstract

**Background:**

Hospital acquired (HA)gram negative bacteremia contribute to substantial mortality and morbidity. Understanding the epidemiology of this entity including susceptibility patterns can inform appropriate antimicrobial therapy.

Objective: To describe the epidemiology, antimicrobial susceptibilities and outcomes of hospital acquired gram negative blood stream infection over six-year period in a quaternary care hospital.

**Methods:**

We used the Infection Prevention Control data base to identify all cases of hospital acquired -gram negative bacteremia from Jan 1 2014 to Dec 31 2019. Charts and susceptibilities were reviewed retrospectively.

**Results:**

440 HA-gram negative bacteremia cases with 469 gram negative bacterial isolates were included in the study after the exclusion of repeat positives within the first 2 weeks of first positive culture. 65% were male. The mean patient age was 62. Mortality was 23% at 30 days. 90% were monomicrobial infection. The majority had secondary blood stream infection accounting for 80% while 20% had primary blood stream infections. The most common source of secondary blood stream infections was UTI at38%. E. coli was the most common organism isolated during this period followed by K. pneumoniae and P aeruginosa, 44%, 16% and 11% respectively. 14% of isolates were ESBL/AMPC organisms with increasing rates over time. Only 77% of P. aeruginosa was susceptible to meropenem. 42%(n=22/52) of Pseudomonas isolates were multidrug resistant and 12%(n=6/52) were identified as difficult to treat resistance. Enterobacterales group organisms (n=372) were susceptible to ceftriaxone 82%, meropenem 99%, piperacillin-tazobactam 80%, ciprofloxacin 77% and TMP-SMX 74%. Overall, vast majority of gram-negative isolates were susceptible to meropenem (97%).

**Conclusion:**

Over the six-year period there was a slight increase in ESBL/AMPC organisms but resistance of non ESBL organisms remained stable. The majority were susceptible to meropenem (97). A significant percentage of P. aeruginosa was resistant to meropenem (23%).

**Disclosures:**

**All Authors**: No reported disclosures

